# AXL/Gas6 signaling mechanisms in the hypothalamic-pituitary-gonadal axis

**DOI:** 10.3389/fendo.2023.1212104

**Published:** 2023-06-15

**Authors:** Pardis Mohammadzadeh, Gregory C. Amberg

**Affiliations:** Department of Biomedical Sciences, Colorado State University, Fort Collins, CO, United States

**Keywords:** TAM receptor tyrosine kinase, hypothalamus, anterior pituitary, gonads, signaling/signaling pathways

## Abstract

AXL is a receptor tyrosine kinase commonly associated with a variety of human cancers. Along with its ligand Gas6 (growth arrest-specific protein 6), AXL is emerging as an important regulator of neuroendocrine development and function. AXL signaling in response to Gas6 binding impacts neuroendocrine structure and function at the level of the brain, pituitary, and gonads. During development, AXL has been identified as an upstream inhibitor of gonadotropin receptor hormone (GnRH) production and also plays a key role in the migration of GnRH neurons from the olfactory placode to the forebrain. AXL is implicated in reproductive diseases including some forms of idiopathic hypogonadotropic hypogonadism and evidence suggests that AXL is required for normal spermatogenesis. Here, we highlight research describing AXL/Gas6 signaling mechanisms with a focus on the molecular pathways related to neuroendocrine function in health and disease. In doing so, we aim to present a concise account of known AXL/Gas6 signaling mechanisms to identify current knowledge gaps and inspire future research.

## 1 Introduction

AXL belongs to the TAM family of signaling proteins formed by a trio of structurally and functionally related receptor tyrosine kinases. Tyro3, AXL, and Mer are widely expressed in immune, cardiovascular, neuronal, and reproductive tissues. AXL was first identified as a human gene of unknown function in 1988 with a screen for transforming genes present in hematogenous cells isolated from patients with chronic myeloid leukemia (1). AXL was subsequently detected by two groups using a transfection-tumorigenicity approach. Janssen et al. isolated a transforming gene, termed UFO to underscore the enigmatic nature of the protein, with predicted structural and functional features consistent with a receptor tyrosine kinase (2). Concurrently, O’Bryan et al. identified the same putative receptor tyrosine kinase, which they called AXL, a derivation from the Greek word anexelekto, meaning uncontrolled (3).

The human *Axl* gene is located on chromosome 19q13.2 and contains 20 exons; alternative splicing results in two transcriptional variants (4). Full length AXL has a molecular weight of 104 kD and post translational glycosylation produces proteins of 120 (partial glycosylation) and 140 kD (full glycosylation). In addition to full-length AXL, a soluble 70-85 kD form resulting from proteolytic cleavage of the extracellular domain and can be detected in plasma (5–7). AXL retains the general receptor tyrosine kinase structure with a ligand binding extracellular N-terminus and an intracellular C-terminus with intrinsic tyrosine kinase activity. As with other TAM receptors, the extracellular portion of AXL is modular and contains two immunoglobulin-like repeats (IgL domains) and two fibronectin type 3-like repeats (FNIII domains) (4). The C-terminus contains six tyrosine residues which are targets for regulatory phosphorylation. The three distal tyrosines near the C-terminus function as docking sites for intracellular signaling proteins while the three proximal sites are involved with regulation of intrinsic AXL tyrosine kinase activity (8).

## 2 AXL activation

**Figure 1:**
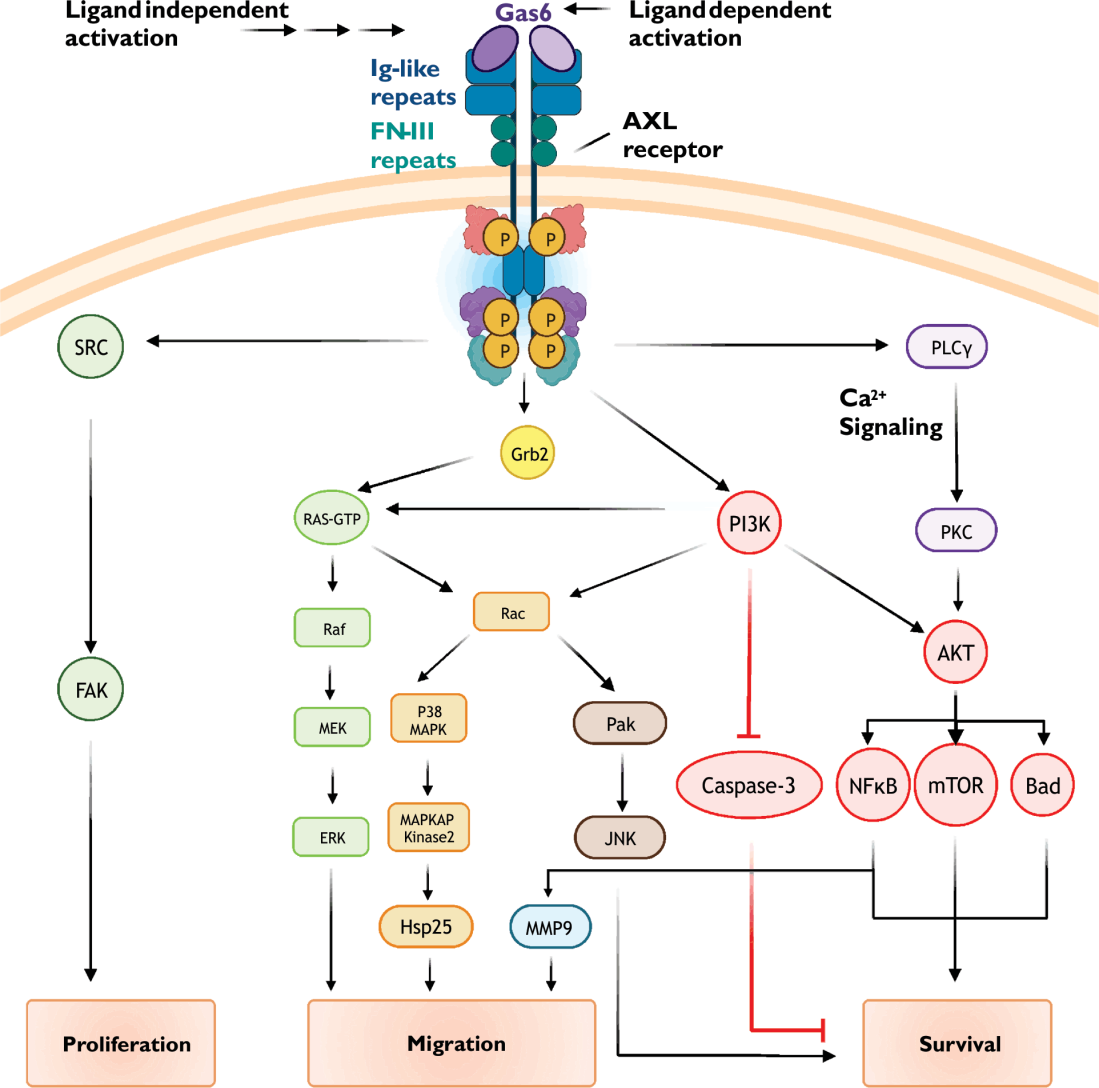
Common AXL receptor signaling mechanisms. AXL receptor activity is coupled to multiple signaling pathways including PI3K/Akt, small GTPase, and MAP kinase cascades. See text for details.

First identified in serum-starved fibroblasts, growth arrest-specific protein 6 (Gas6) is recognized as the primary endogenous AXL receptor agonist (7, 9). Gas6 is a vitamin K-dependent protein structurally related to the anticoagulant Protein S, and contains two laminin-like globular domains (LG1 and LG2) that bind with high affinity to the extracellular immunoglobulin-like repeats (IgL1 and IgL2) of the AXL receptor (10, 11). Gas6 binds to AXL with a 1:1 stoichiometry to promote receptor dimerization and trans-autophosphorylation of regulatory tyrosine residues (**Figure 1**). Tyrosine phosphorylation in turn activates multiple intracellular signaling cascades including those involving Ras, PI3K, PLC-γ, and MAP kinase. Although GAS6 activates each of the three TAM receptors, Gas6 binds to AXL with greater affinity than Tyro3 and Mer (rank order of affinity: AXL>Tyro3>Mer) (12).

In addition to canonical Gas6-dependent activation, several lines of evidence support the concept of ligand-independent AXL receptor activation (13). AXL overexpression increases the probability of receptor-activating homotypic interactions occurring between extracellular domains located on the surface of adjacent cells (14). Similarly, overexpression facilitates ligand-independent homodimerization and activation of receptors expressed in a single cell (15). Receptor activation is also shown to be increased following AXL heterodimerization with non-TAM receptor tyrosine kinases (16, 17).

## 3 AXL expression

Although AXL is widely expressed, the pattern of expression is heterogeneous as AXL is known to be enriched in in several tissues and cell types (4). The molecular determinants of AXL expression are complex and occur at multiple levels including transcriptionally, post-transcriptionally, and pre- and post-translationally. Consistent with its initial discovery as a transforming protein, inappropriate AXL expression (e.g., overexpression, ectopic expression) has been observed in a variety of common cancers (18).

Analyses of regulatory elements in the *Axl* promotor reveal several functional transcription factor binding sites. Examples include YAP/TEAD, Sp1/3, CREB, HIF1α, MZF-1, and AP-1, all of which enhance AXL transcription (19–25). Complementing regulation of AXL expression by transcription factors, AXL expression is suppressed by miRNAs (26) and by methylation of CpG islands located in the *Axl* promotor (23). Given the diversity of regulatory elements influencing AXL transcript levels, it is likely that regulation of AXL expression is highly tissue and cell specific.

Post-translational mechanisms regulating AXL protein stability impact cellular AXL functionality. In addition to tyrosine phosphorylation, Gas6 activation of AXL promotes ubiquitination by the E3 ubiquitin ligase Cbl-b; ubiquitination in turn targets the activated AXL for lysosomal degradation (27, 28). AXL also undergoes presenilin-dependent regulated intramembrane proteolysis (29).

## 4 AXL signaling

### 4.1 Overview

AXL is widely expressed and is coupled to numerous divergent intracellular signaling pathways (19). Similar to determinants of expression, the biological consequences of AXL receptor activation are largely tissue and cell-type specific. In healthy cells, AXL receptor signaling is associated with cell survival, proliferation, migration, and adhesion. In transformed cells, these cellular effects are misappropriated to promote abnormal cell growth, epithelial-mesenchymal transition, metastasis and invasion, angiogenesis, immune evasion, and drug resistance.

Following receptor activation, intrinsic kinase activity-dependent phosphorylation of key tyrosine residues in the AXL C-terminus (Y779, Y821, and Y866) promotes subplasmalemmal recruitment of signaling proteins *via* generation of docking sites for specific protein-protein interactions (**Figure 1**) (30–33). When phosphorylated, Y821 binds proteins containing SH-2 domains including the regulatory phosphatidylinositol 3-kinase (PI3K) subunit p85, SRC cellular tyrosine kinase (c-SRC), and phospholipase C-γ. Phosphorylated Y821 also binds the SH-2 containing growth factor receptor-bound protein GRB2, an adaptor protein which promiscuously couples receptor tyrosine kinases to multiple signaling cascades such as RAS/RAF/MAPK and the Rho GTPase Rac1. To further diversify signaling outputs, AXL clusters and functionally interacts with other receptor tyrosine kinases including EGFR (epidermal growth factor receptor) and HER2 (human epidermal growth factor receptor 2) (34, 35)

### 4.2 PI3K/Akt signaling

PI3K/Akt signaling cascades stimulate a diverse array of cellular processes including protein synthesis, metabolism, and motility. Activation of numerous receptors, including G-protein coupled receptors and notably receptor tyrosine kinases such as AXL, increase PI3K/Akt signaling to promote growth, proliferation, and survival (36, 37). In numerous healthy cells such as vascular smooth muscle and endothelial cells (36, 38), hepatic stellate cells (39), and neurons (40), AXL-dependent PI3K/Akt signaling protects against apoptosis. Many cancer cells, including chronic lymphocytic leukemia (41), breast and prostate carcinomas (42, 43), and non-small cell lung cancers (44), show AXL-dependent PI3K/Akt signaling which drives proliferation, metastasis, invasion, and drug resistance.

AXL activation, either through ligand binding (i.e., Gas6) or ligand-independent mechanisms, promotes the recruitment of p85, the regulatory subunit of the lipid kinase PI3K. PI3K phosphorylates phosphatidylinositol 4,5-bisphosphate (PIP_2_) to form phosphatidylinositol trisphosphate (PIP_3_) (45). Increased abundance of PIP_3_ increases the activity of the serine/threonine kinase Akt/protein kinase B (PKB). Akt substrates include several pro-survival proteins such as MDM2, IKK, and mTOR (46, 47). Phosphorylation of these proteins leads to inactivation of pro-apoptotic signalling proteins (e.g., BAD, a Bcl-2 family member) and apoptosis executing proteins (e.g., caspases 3 and 9).

Akt activity promotes nuclear translocation of the transcription factor NF-kB (48). NF-kB increases the expression numerous cell survival genes including those encoding for anti-apoptotic proteins (e.g., Bcl-xL and Survivin), cell cycle regulators (e.g., Cdk2), proliferation associated proteins (e.g., CD44), and other pro-survival transcription factors (e.g., Twist, Snail, and Slug). NF-kB also increases the expression of matrix metalloproteins, such as matrix metalloproteinases 2 and 9 (MMP2 and MMP9), which alters cell migration, proliferation, and survival (49, 50).

### 4.3 Small GTPase signaling

Small GTPases belonging to the Ras superfamily of signaling proteins are frequent targets of tyrosine kinase receptors. RhoA, Rac1, and Cdc42 are the most widely expressed and characterized members of the Rho family of small GTPases. These proteins contribute to actin cytoskeletal remodeling and formation of cellular protrusions (51). RhoA is usually associated with focal adhesions and stress fiber formation, Rac1 with lamellipodia extension, and Cdc42 with filopodia generation.

Small GTPase-dependent effects of AXL receptor activation have been attributed to Rac1 and RhoA (37, 52). Mechanisms regulating Rac1/RhoA activity include Ras-dependent and independent PI3K/Akt signaling. Interestingly, evidence suggests that AXL function and downstream signaling is subject to oxidant-dependent regulation (53). NADPH oxidase, a major source of reactive oxygen species, is a well-characterized effector of Rac1 and PI3K/Akt (54–56). Downstream effectors of Rac1/RhoA include PAK/JNK and MAP kinase signaling cascades; the general outcome of these signaling events is reorganization of the actin cytoskeleton and cell movement and migration (57–59).

### 4.4 MAP kinase signaling

Mitogen-activated protein kinases (MAPK) transduce extracellular signals from a variety of receptors into changes in fundamental cellular processes ranging from proliferation and survival to migration and differentiation. AXL receptors are known to couple to the extracellular signal-regulated kinase (ERK) branch of MAPK signaling pathways (60, 61). In healthy cells, such as chondrocytes, vascular smooth muscle cells, and GnRH neurons, AXL-dependent ERK signaling promotes cell survival, proliferation, and differentiation (19). In cancer cells, these AXL-dependent signaling mechanisms are hijacked and associated with poor clinical outcomes due to loss of apoptotic potential and resistance to therapy.

### 4.5 AXL signaling summary

AXL receptor activation is complex as it can involve ligand-dependent (e.g., Gas6) and ligand-independent mechanisms. The complexity of AXL signaling continues with the substantial crosstalk between redundant and nonredundant signaling cascades (see **Figure 1**). Given the large number of signaling protein interactions and combinatorial possibilities, it is likely that the cellular effects of AXL activation are going to differ between cell types due to differential expression of specific participating proteins (33). Similarly, as exemplified in transformed cells, AXL-dependent cellular functions can change over time as proteomic landscapes continually evolve. Adding yet another layer of complexity is the poorly understood functional relationship between AXL and other distantly receptor tyrosine kinases such as EGFR (epidermal growth factor receptor) (16).

## 5 AXL/Gas6 function in the reproductive system

The hypothalamic-pituitary-gonadal (HPG) axis regulates and produces the hormones involved with sexual differentiation and reproduction. HPG axis signaling begins when secretory hypothalamic neurons secrete gonadotropin releasing hormone (GnRH) into the hypothalamic-hypophysial-portal circulation. GnRH subsequently binds to and activates GnRH receptors on the surface of gonadotrope cells located in the anterior pituitary (adenohypophysis). Correct cellular location and positioning of GnRH neurons is essential for communication between the spatially disparate components of the hypothalamus and anterior pituitary.

AXL/Gas6 signaling is essential for the proper development of hypothalamic GnRH neurons. Cells destined to become hypothalamic GnRH neurons are formed in the olfactory placode and migrate to the forebrain (62). AXL contributes to this process by promoting Rac1-dependent chemotactic migration of the developing GnRH neurons (52). In immortalized migratory GnRH neurons, Rac1 activity is essential for actin cytoskeletal remodeling and migration following Gas6-dependent AXL activation. Rac1 was found to increase p38 MAPK/MAPKAP kinase 2 activity leading to phosphorylation of HSP25, a small heat shock protein known to interact with actin and intermediate filaments to regulate actin polymerization and cell movement. Additionally, AXL-dependent ERK and PI3K/Akt signaling increases the survival of the migrating cells through anti-apoptotic mechanisms (40). In immortalized non-migratory neurons, AXL signaling suppresses GnRH expression (63). Suggesting signaling complexity, as with GnRH neuron migration, Rac1 was also involved with AXL-dependent inhibition of GnRH production.

In addition to sharing a common ligand (Gas6), the three TAM receptors (Tyro3, AXL, and Mer) exhibit considerable structural homology and functional redundancy (64). Accordingly, investigations of AXL receptor biology are often complicated by potential confounding influences from Tyro3 and Mer receptors. As an approach to account for confounding influences from the trio of TAM receptors, mice expressing different combinations of TAM receptor null mutations were generated (65). Initial characterization revealed that only TAM receptor triple knockout mice displayed an obviously abnormal reproductive phenotype.

Subsequently, AXL/Tyro3-null (double knockout) mice revealed clear, albeit nuanced, roles for these two TAM receptors in female reproductive function (66). Confirming earlier findings (40, 63), GnRH neuronal survival and migration were impaired in female AXL/Tyro3-null mice. Compared to wild-type controls, female AXL/Tyro3-null mice showed delayed time to first estrous. Although the onset of vaginal opening was found to be normal, AXL/Tyro3 knockout was associated with abnormally prolonged estrous cycles. Ovaries from AXL/Tyro3-null mice were histologically unremarkable. AXL/Tyro3-null females also showed normal pituitary responses (i.e., increased luteinizing hormone production) and sensitivity to exogenous GnRH (67). In contrast, ovariectomized AXL/Tyro3-null mice failed to respond to exogenous estradiol suggesting that impaired GnRH neuronal function. Altogether, these observations suggest that AXL/Tyro3 signaling could contribute to the cyclicity and normal female reproductive function.

All three TAM receptors (Tyro3, AXL, and Mer) are thought to contribute to normal male reproductive function (65). Specific roles for the individual members have been obscured by the experimental constraints associated with the complicated functional relationship between the three receptors. However, male TAM receptor triple knockout mice were sterile, and only produced immature sperm as a result of the progressive death of differentiating germ cells (68). The effects of TAM receptor knockout were only detected postnatally; embryonic and neonatal testes were not obviously affected. Although reported expression patterns vary, Tyro3, AXL, and Mer, as well as Gas6, are known to be differentially expressed in testicular somatic support cells of the testes (e.g., Leydig and Sertoli cells) and spermatogonia (65, 68–70). Expression discrepancies likely stem from differences in measured parameters (e.g., transcript versus protein), cell type studied (e.g., native tissue verses cell lines), and temporal/developmental considerations (e.g., perinatal versus postnatal). Interestingly and consistent with a self-regulating feedback mechanism, Gas6, perhaps produced by Leydig cells, was found to be upregulated in TAM triple knockout mice (65, 69, 70).

Observations from TAM triple knockout mice indicate that the three receptors work cooperatively to support spermatogenesis. Of the supportive somatic cells, Sertoli cells appear to be the most impaired in TAM triple knockout mice and Leydig cells the least affected. Sertoli cells from TAM triple knockout and Mer-null mice showed dysfunctional phagocytic activity while cell survival and differentiation appeared to be unaffected (70, 71). Spermatogenesis was more severely impaired in TAM triple knockout mice than mice lacking only Mer receptors. Interestingly, AXL receptor expression was found to be upregulated in Mer-null mice (71). Impaired phagocytotic function in Sertoli cells negatively impacts spermatogenesis, likely by removing apoptotic cells and increasing nutrient availability (72–74).

## 6 Reproductive disorders associated with AXL

Although AXL is associated with the development of a variety of cancers, *Axl* mutations are rarely identified as causative and AXL appears to contribute to cancer development through mechanisms involving increased levels of expression (19, 22, 75). Consistent with the proposed role of AXL in cancer, *Axl* mutations and dysfunctional AXL protein are not widely implicated in the development of reproductive disorders. However, AXL dysfunction has been linked to some forms of Kallmann Syndrome, a subtype of idiopathic congenital hypogonadotropic hypogonadism.

Evidence suggests that rare variants of idiopathic hypogonadotropic hypogonadism can be associated with *Axl* mutations that impair AXL function (76). Idiopathic hypogonadotropic hypogonadism involves defects in neuronal GnRH release or GnRH action (77, 78). Consistent with the documented role of AXL in GnRH neuronal migration from the olfactory placode to the hypothalamus, these mutations result in impaired survival and migration of neurons, including GnRH neurons, from the medial olfactory placode to the hypothalamus. The consequences of diminished hypothalamic GnRH neuronal populations associated with low levels or lack of circulating testosterone, luteinizing hormone, and follicular stimulating hormone (FSH).

Altogether, these observations suggest that altered function of AXL, and other TAM receptor components (e.g., Tyro3, Mer and Gas6), can contribute to endocrine dysfunction and disease.

## 7 Conclusions and future directions

**Figure 2:**
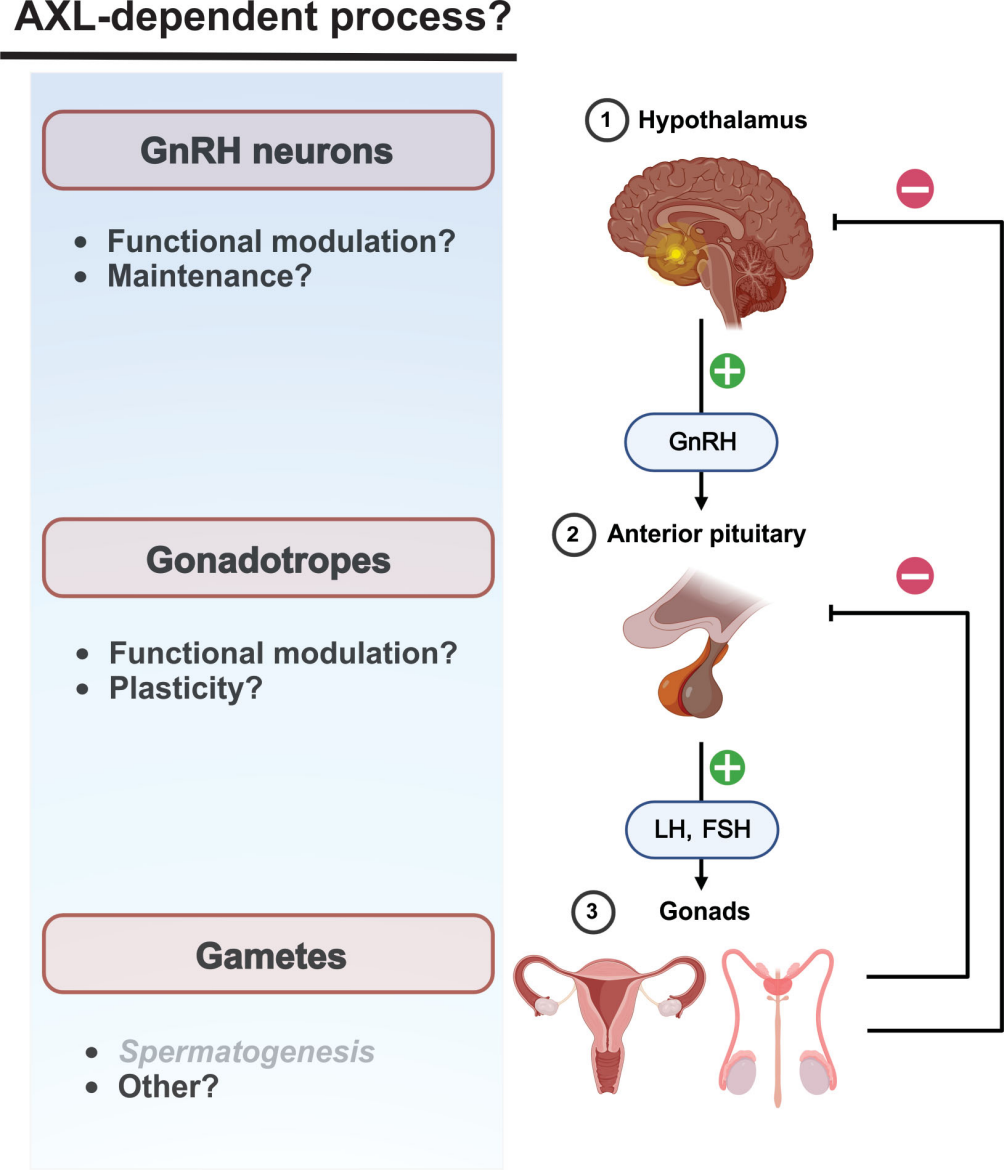
Neuroendocrine AXL/Gas6 functions are poorly characterized post-developmentally. Potential influence of AXL/Gas6 signaling post-developmentally along he HPG axis. AXL/Gas6 support of spermatogenesis is clearly established.

As outlined above, AXL receptors serve as signaling hubs coupled to a variety of fundamental cellular processes including survival, proliferation, and migration. A clear understanding of the importance of AXL/Gas6 contributions to neuroendocrinological function in health and disease is lacking (**Figure 2**). Indeed, the pattern and extent of AXL expression in adult neuroendocrine tissues is largely uninvestigated. The complexity of AXL functional interactions with other TAM receptors as well as more distantly related receptor tyrosine kinase receptors (e.g., EGFR, PDGF) creates a daunting experimental challenge that is not easily overcome. However, these very complexities underline the necessity and motivation for further investigation of AXL receptor biology.

Hypothalamic kisspeptin neurons are upstream regulators of GnRH neurons and mediate estradiol-dependent feedback control of gonadotropin generation. Activation of presynaptic and postsynaptic kisspeptin receptors (Kiss1R) on kisspeptin and GnRH neurons, respectively, are critical for physiological patterns of gonadotropin generation including the estradiol-dependent LH surge preceding ovulation (79). Of potential interest, kisspeptin increases transcriptional expression of AXL in some cancers (80, 81). Although a role for AXL in the post-developmental hypothalamus has not been described, kisspeptin-modulated AXL function could contribute to maintenance and modulation of synaptic communication between kisspeptin and GnRH neurons.

AXL receptor promotion of cell movement could contribute to neuroendocrine function beyond GnRH neuron migration during development. Evidence suggests that gonadotropes in the anterior pituitary move towards the hypophyseal portal capillaries, possibly to facilitate the transfer of gonadotropins into the blood (82, 83). However, the molecular mechanisms underlying GnRH receptor-dependent changes in gonadotrope chemotaxis and plasticity are unclear. Although the functional significance has not been addressed, AXL transcripts have been detected in mouse gonadotropes (84).

With the expanding role of AXL to the development of cancer, AXL has been identified as a promising candidate for novel targeted chemotherapeutic agents (61, 85). No fewer than ten AXL receptor inhibitory compounds are under clinical investigation for the treatment of many cancers, including neuroendocrine-related tumor of the breast, ovary, and pancreas (86). These investigational agents include small molecule inhibitors, monoclonal antibodies, antibody-cytotoxic drug conjugates, CAR-T cell therapeutics, and soluble AXL receptor fusion proteins.

Note that some of these agents inhibit AXL receptor signaling and others use AXL to target cytotoxic agents to specific populations of cells.

The consequences of these disparate mechanisms make predicting and addressing off-target effects of AXL-directed chemotherapeutics challenging in the absence of information regarding AXL receptor expression and function post-developmentally (**Figure 2**). As described above, the importance of AXL signaling to HPG function in adults is generally unknown at present. Given the functional redundancy of TAM receptors, the consequences of therapeutic AXL receptor inhibition will depend not only on the degree of AXL function, but also the coexpression and activity of Tyro3 and Mer. In contrast, AXL-targeted cytotoxic therapeutics could have unexpected consequences not observed with conventional AXL receptor inhibition. Accordingly, improved understanding of AXL expression and function in the reproductive system is warranted.

## Author contributions

Conceptualization, PM; Writing and literature review, PM and GA; Critical revision of the manuscript, GA. All authors contributed to the article and approved the submitted version.
